# Awareness of Child Sexual Abuse and the Protection of Children From Sexual Offences Act Among Medical and Paramedical Students in Vidisha, Madhya Pradesh

**DOI:** 10.7759/cureus.111526

**Published:** 2026-06-26

**Authors:** Pratik Sahu, Richa Nigam, Vipul Agrawal, Kasthala Mounika, Sanjay S Agarwal

**Affiliations:** 1 Department of Community Medicine, Atal Bihari Vajpayee Government Medical College, Vidisha, IND

**Keywords:** awareness, child sexual abuse, medical education, pocso act, public health

## Abstract

Background: Child sexual abuse (CSA) remains a pervasive public health and human rights concern in India, with healthcare professionals occupying a critical position in early identification, reporting, and legal compliance. Adequate awareness of CSA and familiarity with the Protection of Children From Sexual Offences (POCSO) Act remain essential for effective child protection.

Objectives: This study assessed awareness regarding the status of CSA and evaluated knowledge of the POCSO Act among medical and paramedical students in a government medical college in central India.

Methods: A cross-sectional, questionnaire-based study was conducted among 278 medical and paramedical students. A validated, structured tool comprising two sections assessed awareness of CSA and knowledge of the POCSO Act. Responses were scored and categorized into predefined levels. Descriptive statistical analysis was performed.

Results: Awareness of CSA remained inadequate across both groups, with no participant demonstrating complete awareness. Most medical students exhibited inadequate awareness, while a substantial proportion of paramedical students remained unaware. Knowledge of the POCSO Act was predominantly moderate, with medical students demonstrating comparatively better understanding than paramedical students. Gaps were evident in knowledge related to mandatory reporting, legal procedures, and disclosure dynamics, despite high recognition of the Act’s existence.

Conclusion: The findings reveal significant deficiencies in awareness and legal preparedness among future healthcare professionals. Integrating structured, practical, and legally oriented training on CSA and the POCSO Act within medical and paramedical curricula is imperative to strengthen early detection, reporting, and child protection outcomes.

## Introduction

Child sexual abuse (CSA) is a serious violation of the basic human rights of children and remains a chronic global public health issue. It can be described as the sexual involvement of a child by someone in a position of authority, trust, or power, such as a parent, family member, elder sibling, caregiver, or another responsible adult, under conditions in which the child is psychologically immature or below the legal age to provide informed consent [1]. It may occur in various environments, such as homes, schools, workplaces, and the community, and is often concealed due to fear, stigma, social norms, and an unwillingness to reveal it [2]. CSA has long-term effects on mental health, emotional development, and social functioning, in addition to its immediate impact.

The national surveys and regional studies have reported the scale of CSA in the Indian context [3]. In another historic study carried out in 2007 by the Ministry of Women and Child Development, researchers concluded that about 53% of Indian children had suffered some form of sexual abuse in childhood. Officially recorded cases have shown a steadily increasing trend over the last 10 years, whereby registered cases increased to 14000 in 2015 compared to 8804 in 2014. Data from the region also underline that the problem is widespread. A survey conducted in Kerala indicated that 35% of boys and 36% of girls below 18 years of age had experienced sexual abuse. Children in vulnerable categories, such as street children, those in institutional care, child laborers, and children between five and twelve years, are especially vulnerable [4]. India is still one of the nations with a high incidence of sexual crimes, which underscores the urgent need for effective preventive, clinical, and legal measures [5].

There are serious and complex health implications of CSA. Patients bringing their children to an emergency department or a healthcare facility without proper assessment are at higher risk, and it is reported that 5%-10% may die and 35%-50% may sustain severe injuries if they do not receive timely treatment [6]. In addition to short-term physical injuries, survivors often have long-term psychological sequelae, such as depression, anxiety disorders, behavioral disturbances, impaired self-esteem, and problems with social adjustment [7]. These findings highlight the importance of healthcare professionals in the early detection of suspected cases of CSA, proper registration, and prompt handling of such cases.

The Government of India has recognized the increasing burden of CSA and, on 14 November 2012, enacted the Protection of Children From Sexual Offences (POCSO) Act [8-10]. This comprehensive legislation seeks to safeguard children against sexual abuse and exploitation by providing a well-defined legal framework, child-friendly reporting systems, standardized medico-legal practices, and the establishment of special courts to ensure timely trials [11]. The Act mandates reporting of offences, ensures that the identity of the child remains confidential, and requires that victims be handled sensitively during legal proceedings [12]. Despite these provisions, important elements pertinent to medical practice, including forensic examination, preservation of evidence, documentation of injuries, psychological support, and legal compliance, are poorly integrated into routine medical and paramedical training [13].

Lack of systematic and detailed training has led to a significant lack of awareness and readiness among healthcare trainees [14]. A large number of medical and paramedical students are not aware of subtle clinical signs of abuse, proper reporting procedures, and the medico-legal requirements of child protection legislation [15]. Considering the growing rate of sexual abuse among children, organized education programs in association with academic institutions, healthcare sectors, and implementing authorities are necessary to enhance awareness and response mechanisms [16]. This is because sensitization and training of future healthcare providers are important steps in making early detection, reporting, and comprehensive management of affected children more effective.

It is against this backdrop that it is important to assess levels of awareness and knowledge among medical and paramedical students. These groups are frontline healthcare providers and play a critical role in protecting vulnerable children [17]. The current research was thus conducted to assess awareness of the current situation of CSA in society and to determine the level of knowledge about the POCSO Act among medical and paramedical students [18]. The strengths and weaknesses of current knowledge will help identify gaps in education that can be used to implement specific interventions to improve professional competence, legal compliance, and child protection practices.

Objectives of the study

This study aimed to assess awareness of CSA and to evaluate knowledge of the POCSO Act among medical and paramedical students.

## Materials and methods

Sampling method and study population

In the study, 278 medical and paramedical students from a government medical college in Vidisha, Madhya Pradesh, were involved. Of the respondents, 155 were medical students, and 123 were paramedical students. Convenience sampling was used to recruit participants. Participants were not obligated to take part in the study, and participation was based on informed consent. Students were informed about the purpose of the study, the confidentiality of responses, and their right to withdraw at any stage without any academic consequences. Only students who were present on the day of data collection and were willing to participate were included in the study.

Data collection tool

Data were collected using a validated, structured questionnaire that had two parts, each containing 10 questions. The first part consisted of questions regarding the prevalence of CSA, its consequences on children, and best practices in reporting cases. The second part was based on the POCSO Act and its applicability to various aspects of CSA, such as legal responsibilities, penalties, and procedural management [8]. The questions were in a four-choice format, with each item being an alternative. The participants were advised to make one choice. The questionnaire aimed to evaluate both awareness and practical knowledge relevant to child protection responsibilities among future healthcare providers. Questions were framed in simple language to ensure uniform comprehension among the students.

Data collection procedure

The questionnaire was filled out by the participants at the start of lectures. The standardized instructions allowed 15 minutes, during which participants were required to complete the questionnaire. Students were seated separately to minimize discussion or influence from their peers. One faculty member, along with the principal investigator, was present during data collection to avoid response bias. Completed questionnaires were collected immediately after completion to avoid post-response modification.

Scoring and categorization

An individual question had one mark each, and the highest mark in each part of the questionnaire was 10 marks. Each section was evaluated individually. The students were categorized into three groups based on their scores: unaware (scores 1-4), inadequately aware (5-8), and aware (>8). In the case of POCSO Act knowledge, the same scoring criteria were used to classify the participants as having poor, moderate, and good knowledge. The scoring criteria were adopted from a similar study that was used to assess awareness of CSA and the POCSO Act among medical students [19].

Questionnaire validation

The questionnaire was designed as a multiple-choice tool and was approved by two subject experts. The first part was based on a pre-tested questionnaire that was utilized in another study [19]. The second section, which dealt with other provisions of the POCSO Act, was developed with the help of subject experts to include all domains of the Act. The two-part questionnaire was validated using a pilot study conducted on 30 medical and paramedical students. The questionnaire was available in English and Hindi, and participants were allowed to respond in either language. Necessary linguistic validation was performed to ensure that both language versions conveyed the same meaning.

Data analysis

The data from the completed questionnaires were organized and entered into Microsoft Excel 2019 (Microsoft Corporation, Redmond, WA) and subsequently cleaned. Statistical analyses were performed in accordance with the study objectives. Descriptive statistics, including means, frequencies, proportions, and tables, were used to summarize the study variables. The Chi-square test was applied to assess differences in categorical variables. The significance level was defined at a 95% confidence level, and the results were considered statistically significant when p<0.05. Comparative analyses between medical and paramedical students were performed to assess differences in awareness and knowledge levels. Results were presented in tabular format to facilitate the interpretation and comparison of findings.

## Results

Awareness levels regarding the current status of child sexual abuse

The spread of awareness levels about the state of CSA among medical and paramedical students. The results show that awareness is still, to a large extent, restricted in both groups. There was a significant number of students in each category who displayed a lack of awareness, and the rest of the students fell under the poor awareness category. Notably, none of the respondents in either academic stream demonstrated a sufficient awareness level on the topic, which is why educational intervention and specific sensitization of medical and paramedical educational programs must be enhanced, as depicted in Table 1.

**Table 1:** Level of awareness about the status of CSA CSA: child sexual abuse

S. no.	Awareness level	Medical students, n (%)	Paramedical students, n (%)	Total
1	Unaware	38 (24.52)	38 (30.89)	76
2	Inadequate	117 (75.48)	85 (69.11)	202
3	Adequate	0 (0.00)	0 (0.00)	0
	Total	155 (100)	123 (100)	278

Knowledge of the Protection of Children From Sexual Offences Act

The knowledge levels about the POCSO Act among medical and paramedical students are shown in Table 2. The distribution reveals that the majority of respondents in the two groups had moderate knowledge of the Act. The percentage of students who had good knowledge was lower, and there was a small proportion with poor knowledge. Comparative observation indicates a difference between medical and paramedical students in the level of their knowledge, with medical students having a relatively higher level of knowledge. The table indicates that, despite the level of awareness about the POCSO Act among future healthcare professionals, there is still a gap in legal education and systematic training in order to achieve a broad and profound understanding and application in clinical and professional practice, as represented in Table 2.

**Table 2:** Level of knowledge about POCSO Act Legal source: [8] POSCO: Protection of Children From Sexual Offences

S. no.	Knowledge level	Medical students, n (%)	Paramedical students, n (%)	Total
1	Poor knowledge	17 (11)	16 (13.0)	33
2	Moderate knowledge	102 (65.8)	103 (83.7)	205
3	Good knowledge	36 (23.2)	4 (3.3)	40
	Total	155 (100)	123 (100)	278

Comparative knowledge on child sexual abuse

The findings of the differences in the correct responses of medical and paramedical students on the major issues of CSA are shown in Table 3. The comparison includes knowledge of omitted sexual acts, trends in prevalence, the gender involved, and how the effects of abuse persist. It also indicates differences in perceptions of probable offenders, identification of physical cues, and responses to severe injury and disclosure styles. Disagreements in the level of acquaintance with legal provisions and principles of Indian law and attitudes toward the process of CSA education are also observed. The table illustrates differences in legal, clinical, and social awareness between the two groups of students.

**Table 3:** Comparison of correct responses regarding the status of CSA Legal Source: [8] CSA: child sexual abuse

S. no.	Question	Medical students, n=155 (%)	Paramedical students, n=123 (%)	Chi-square (p-value)
1.	Excluded sexual act	96 (61.93)	32 (26.01)	35.615 (<0.001)
2.	Prevalence estimate	34 (21.90)	15 (12.19)	4.481 (0.03)
3.	Most affected gender	97 (62.58)	101 (82.11)	12.767 (0.003)
4.	Duration of effects	134 (86.40)	100 (81.30)	1.366 (0.242)
5.	Most likely perpetrator	33 (21.20)	63 (51.20)	27.172 (<0.001)
6.	Excluded physical sign	104 (67.09)	76 (61.78)	0.847 (0.35)
7.	Action for serious injury	58 (37.41)	41 (34.14)	0.499 (0.57)
8.	Disclosure rate	7 (4.51)	7 (5.69)	0.198 (0.656)
9.	Relevant Indian law	152 (98.06)	93 (75.60)	33.054 (<0.001)
10.	Education inclusion level	122 (78.70)	100 (81.30)	0.286 (0.592)

Comparative awareness of provisions under the Protection of Children From Sexual Offences Act

Comparative evaluation of the knowledge of medical and paramedical students on the essential provisions of the POCSO Act is shown in Table 4. The table shows differences in comprehension with regard to the source of legislation, requirements associated with mandatory reporting, and identification of the population under protection. Another area of difference is seen in their knowledge of the offences included and excluded by the Act, awareness of persons with the authority to file cases, and understanding of the factors that contribute toward aggravating offences. Additional comparisons were made regarding awareness of punishments for child pornography, procedural protections during statement recording, the timing of medical examinations for the child, and expectations for case disposition, as shown in Table 4.

**Table 4:** Comparison of correct responses regarding the POCSO Act Legal Source: [8] POSCO: Protection of Children From Sexual Offences

S. no.	Question	Medical students, n=155 (%)	Paramedical students, n=123 (%)	Chi-square (p-value)
1.	Establishment year	110 (70.96)	53 (43.08)	22.03 (<0.001)
2.	Min. penalty for non-reporting	77 (49.67)	36 (29.26)	11.75 (<0.001)
3.	Legislation target group	103 (66.45)	45 (36.58)	24.58 (<0.001)
4.	Excluded offense	113 (72.90)	102 (82.92)	4.08 (0.04)
5.	Who can report	132 (85.16)	110 (89.40)	1.10 (0.29)
6.	Aggravating factors	139 (89.60)	100 (81.30)	3.94 (0.04)
7.	Child pornography penalty	95 (61.29)	63 (51.21)	2.83 (0.09)
8.	Statement recording presence	132 (85.16)	93 (75.60)	4.13 (0.04)
9.	Medical exam timing	132 (85.16)	87 (70.73)	8.55 (<0.001)
10.	Case disposal duration	94.19	7.73	217.14 (<0.001)

Comparative knowledge across the Protection of Children From Sexual Offences Act domains

**Figure 1:**
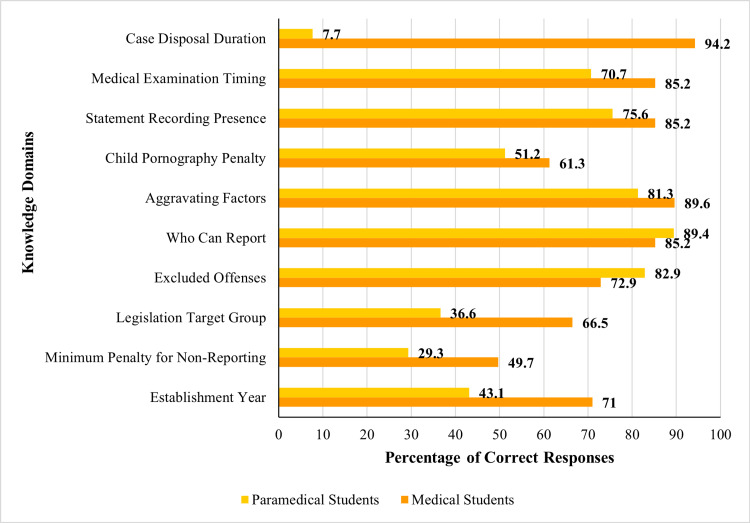
Question-wise awareness of CSA status Legal Source: [8] CSA: child sexual abuse

The differences in medical and paramedical students concerning knowledge of the POCSO Act based on the domain [8]. Among the procedural and legal areas, medical students show higher knowledge of aspects such as the year of establishment, aggravating conditions, recording of statements, timing of medical examination, and duration of case disposal. The performance of paramedical students in excluding the offences and awareness of the reporting authority is relatively higher. Notwithstanding these differences, there are still apparent gaps in both groups in such key areas as penalties and reporting requirements. This type of distribution shows that there is disproportionate knowledge of statutory provisions, which means that the exposure to legal and procedural training is uneven. The number highlights the necessity of having systematic, curriculum-based teaching in order to reinforce domain-related legal literacy, as was depicted in Figure 1.

Domain-wise knowledge patterns among health students

**Figure 2:**
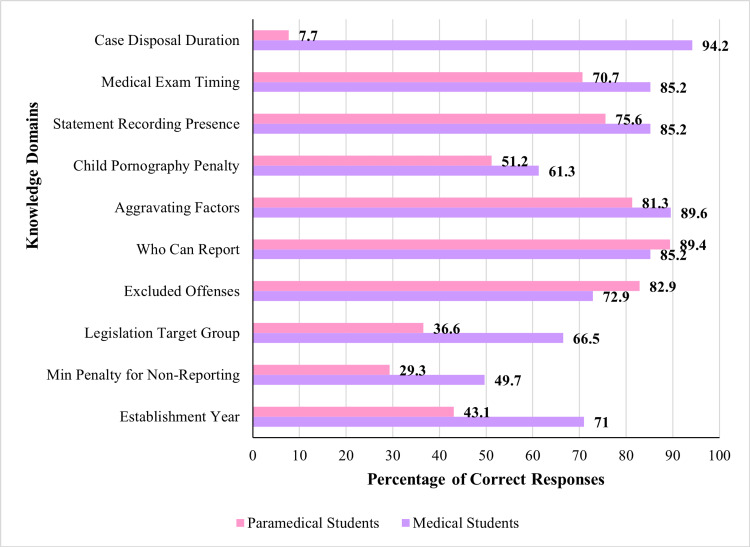
Knowledge of the POCSO Act among students Legal Source: [8] POSCO: Protection of Children From Sexual Offences

The parallel comparison of the knowledge patterns between medical and paramedical students in major areas of the POCSO Act is shown in Figure 2 [8]. Medical students are always more aware in terms of time-bound and enforcement aspects, whereas paramedical students are comparatively stronger in conceptual and definitional aspects. There are areas where the two groups show close agreement, which implies that they have a common baseline exposure. Nonetheless, there are strong variations in some aspects of the law and procedure, denoting fluctuation in the focus of training. The trend is a representation of divided knowledge, as opposed to holistic learning between the two groups. Such results bring about the significance of the need to harmonize the educational materials offered in health fields to have consistent knowledge of child protection laws and their real implications, as presented in Figure 2.

Distribution of awareness levels among medical and paramedical students

**Figure 3:**
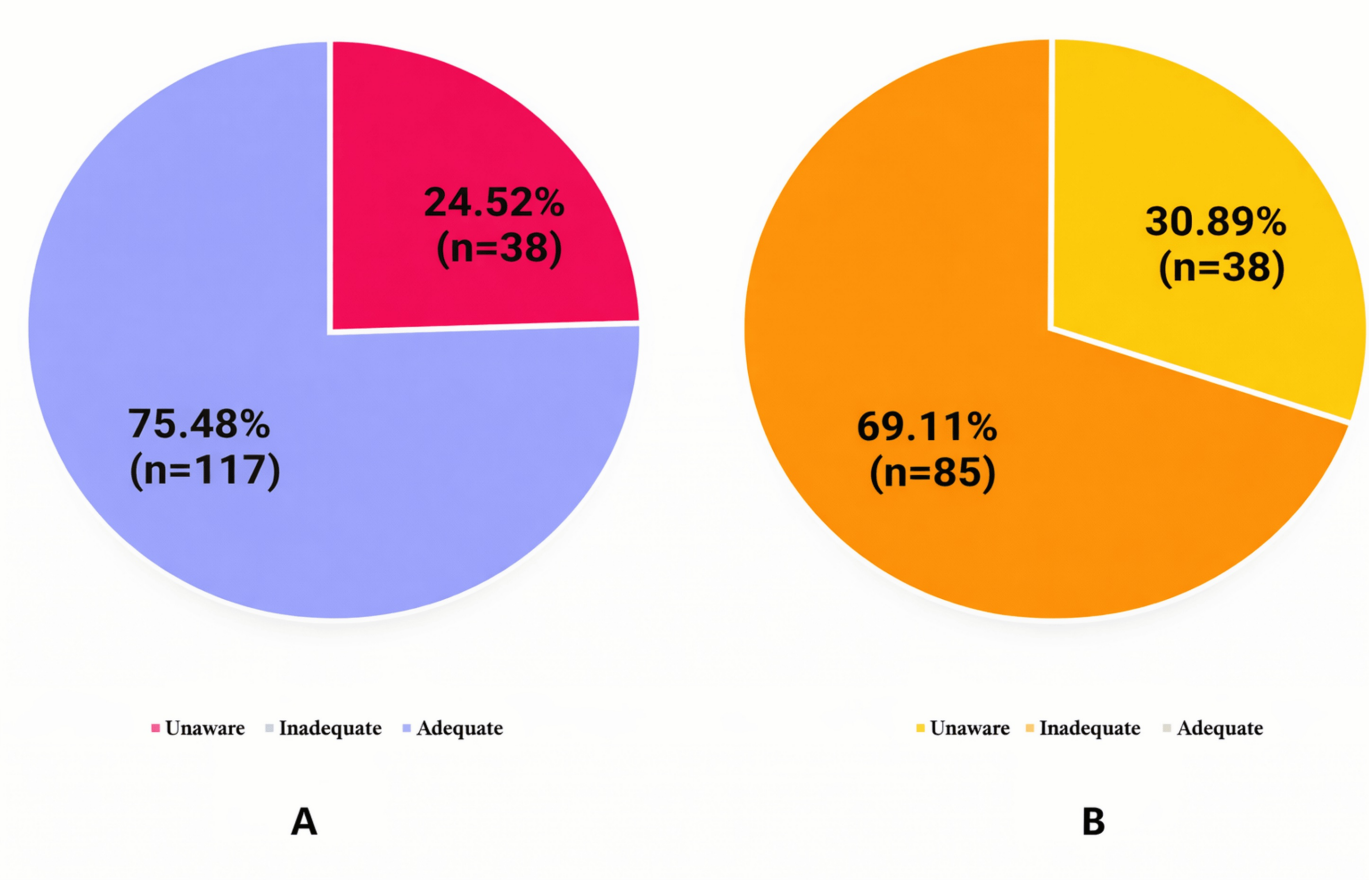
Awareness levels among (A) medical and (B) paramedical students

Figure 3A illustrates the awareness levels of medical students, and there is more inadequate awareness among the students, with a smaller percentage being totally unaware, and no percentage demonstrating adequate knowledge. As Figure 3B shows, the trend among paramedical students is a higher percentage of under-awareness, with a significant level of under-awareness and a smaller percentage at a higher level of understanding. Figures 3A and 3B combined indicate apparent differences between the two groups, where paramedical students were relatively better aware. Nevertheless, both figures together highlight a lack of thorough knowledge, which supports the necessity of systematic educational approaches to increase awareness of CSA and associated legal provisions.

Evaluation of the Protection of Children From Sexual Offences Act knowledge among health science students

**Figure 4:**
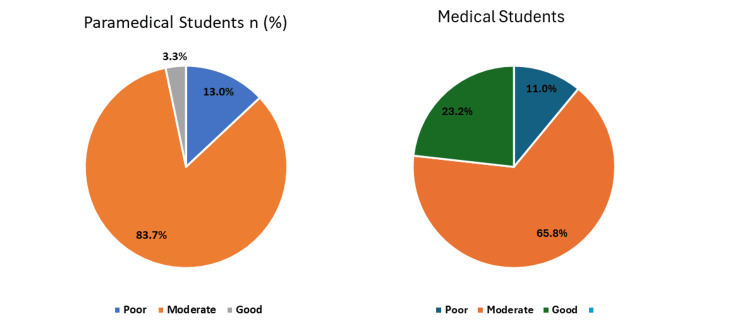
Knowledge of the POCSO Act among (a) medical and (b) paramedical students Legal Source: [8] POSCO: Protection of Children From Sexual Offences

The level of knowledge among medical students is shown in Figure 4A, where most students have moderate knowledge of the POCSO Act, with a small proportion having poor and good knowledge [8]. Figure 4B indicates the trend among paramedical students, in which there is a heavier distribution at the moderate level of knowledge, with fewer students at the extremes. As shown in Figure 4A and Figure 4B, awareness among paramedical students is relatively higher than that of medical students. The two groups do not have strong knowledge. The comparative distribution accentuates partial awareness and not complete understanding, indicating the necessity of rationalized learning programs to enhance legal literacy regarding child protection legislation, as shown in Figure 4.

## Discussion

The study indicates significant areas of awareness and knowledge of CSA and the POCSO Act among medical and paramedical students. The participants were fully aware of the status and size of CSA in India. Most of the students had poor awareness, with 75.48% and 69.11% representing medical and paramedical students, respectively, with the rest of the participants being completely unaware of major CSA facts. These results suggest that even future medical professionals will not be adequately prepared to recognize, handle, and respond to instances of CSA.

The demographic profile of India, where more than 40% of the population is below the age of 18 years, highlights the scale of this issue in terms of public health. According to national data and prevalence studies, a considerable percentage of children are sexually abused in childhood, with estimates between 18% and 50%. The responsibility to identify abuse, ensure the safety of the child, and facilitate necessary care and legal reporting is assigned to healthcare providers in international guidelines. Nevertheless, the current research has shown that awareness among students is still low, as evidenced by previous studies indicating low awareness among medical trainees. According to national crime statistics on CSA victims, girls constitute the vast majority of victims of both penetrative and non-penetrative sexual crimes. The present research found that 62.58% of medical students and 82.11% of paramedical students were able to describe features related to an increased risk of CSA, consistent with earlier reports of widespread abuse in both genders, but higher vulnerability among girls. These results indicate that there is partial knowledge of risk profiles, but this information is not always translated into complete knowledge and appropriate action.

The physical, psychological, and social implications of CSA are long-term in nature. Survivors tend to exhibit increased levels of depression, anxiety, mood disorders, disrupted self-esteem, and physical conditions such as sleep disturbances, asthma, and frequent infections [19]. Investigations have also shown that individuals who have experienced adverse childhood experiences have heightened suicidal thoughts. The persistence of these effects underscores the significance of early detection and intervention by medical practitioners, and therefore the necessity to strengthen training at the undergraduate level [20]. In line with previous research, the present study found that offenders are most often familiar to the victim and frequently occupy positions of authority [21]. The knowledge of the profiles of perpetrators, however, differed significantly across the participants. A proportion of students identified known individuals or family members as frequent offenders, while a large percentage still associated CSA mostly with strangers or were unsure. This misconception can contribute to delayed prevention and postpone preventive measures.

Clinical signs and symptoms of CSA were recognised significantly better, and more than half of both medical and paramedical students responded correctly to the distinction between CSA-related clinical signs and symptoms and others. This is a step forward compared to previous research, which indicated low diagnostic awareness among medical students [22]. Loopholes still exist, especially in the application of recognition in corresponding medico-legal action. It is important to note that a few students claimed that they would immediately inform law enforcement when they encounter a child with severe injuries, being unaware of their duty to report under child protection laws [23].

There was also limited knowledge associated with disclosure patterns and reporting mechanisms. Even though there is evidence indicating that a significant percentage of children report abuse when offered a safe environment, only a small percentage of students can correctly report disclosure rates [24]. Conversely, both groups were highly aware of the existence of the POCSO Act, with awareness levels higher than those reported in previous studies [8,25,26]. This difference points to the fact that students might know the law in theory, but their knowledge of how to apply it in practice is not sufficient.

Concerning knowledge about the POCSO Act, the majority of students showed moderate-level knowledge, and only a small percentage showed good knowledge of procedural and legal specifics [8]. The differences noted between medical and paramedical students may be due to differences in the focus of the curriculum, as medical education exposes students to more medico-legal concepts. The need to remedy this imbalance by undertaking specific curricular changes, especially in paramedical training, is necessary to achieve consistency in competency across various healthcare disciplines. To sum up, the current research highlights the necessity of enhancing teaching on CSA and child protection legislation among medical trainees. It is essential to incorporate practical, structured, and legally focused training into medical and paramedical curricula to enhance early identification, correct reporting, and multidisciplinary child protection initiatives. Improved training will better prepare future healthcare workers to serve endangered children as required.

Limitations and future directions

This research has some limitations that ought to be taken into consideration when interpreting the results. In a cross-sectional design, awareness and knowledge are captured at one point in time and do not provide an opportunity to evaluate changes after training or educational intervention. Since the data were gathered using self-reports, the threat of response bias cannot be ruled out, as participants may have rated their knowledge or level of awareness higher than it actually is. Also, the research was carried out in a narrow geographical setting, which may limit the extrapolation of the findings to other areas or healthcare facilities with different training frameworks and exposure. The lack of qualitative assessment also limits understanding of attitudes, perceptions, and barriers to the reporting and management of CSA cases in greater depth.

Further studies are needed using longitudinal and intervention-oriented designs to determine the effectiveness of structured workshops, curricular integration, and continuing medical education programs in enhancing knowledge, legal awareness, and reporting practices among medical and paramedical students. Practical simulations, case-based discussions, and medico-legal training can be incorporated to increase skill retention and confidence in handling suspected cases. Further research across various institutions and regions would provide a broader understanding of the level of preparedness in a country. Moreover, future interventions should be multidisciplinary and community-based, involving healthcare professionals, instructors, and social services to enhance early detection, reporting, and the recovery of sexually abused children.

## Conclusions

This study indicates that there are significant knowledge gaps in awareness and actual knowledge of the POCSO Act among medical and paramedical students, who are among the main stakeholders in child protection. Although awareness of CSA existed at a baseline level, there was a significant area of concern regarding knowledge of the law, mandatory reporting procedures, and medico-legal liability, which may result in underreporting and delayed intervention. Such lapses indicate a gap between theoretical knowledge and practical legal understanding, and necessary steps should be taken by administrators to sensitize future healthcare workers on this topic.

## Appendices

Questionnaire

Part 1: Child Sexual Abuse Awareness Study

Name (Optional):                           Age: Gender: Batch: Residence: 

Guidelines: A. Please tick only one option. B. CSA stands for Child Sexual Abuse.

1) Which of the following does not come under Child Sexual Abuse? a) Sexual assault b) Transsexualism c) Exhibitionism d) Forcible kissing 2) How many children in India do you think are sexually abused? a) 1 in 2 children b) 1 in 10 children c) 1 in 100 children d) Don't know 3) Which gender do you think is more likely to suffer from Child Sexual Abuse in India? a) Males b) Females c) Both sexes are affected equally d) Don't know 4) Without intervention, how long do you think the effects of CSA generally last? a) A few months b) A few years c) Lifelong d) Don't know 5) Who do you think is most likely to sexually abuse a child? a) A stranger b) An acquaintance c) A trusted relative d) Don't know 6) Which of the following do you think is not a sign of CSA? a) Genital pain b) Social withdrawal c) Vomiting d) Bedwetting 7) If you found out that a child has been sexually abused and has serious injuries, what would you do? a) Contact an NGO/helpline b) Inform the police c) Bring the child to a government hospital d) Keep quiet and do not tell anyone 8) Out of the total number of children who have been sexually abused, how many would tell anybody about their experience? a) 0 to 25% b) 26 to 50% c) 51 to 75% d) Don't know 9) Which of the following is the law on CSA in India? a) Child Sexual Abuse Prevention Act b) Protection of Children from Sexual Offences Act (POCSO) c) Child Welfare Act d) Sexual Offences Act 10) From what level do you think information about CSA should be included in our education system? a) Primary school b) Secondary school c) Degree college for concerned professionals (e.g., doctors, teachers, etc.) d) Should not be included

Part 2: POCSO Act

1) When was the POCSO Act established? a) 2019 b) 2018 c) 2012 d) 2010 2) According to the POCSO Act, what is the minimum punishment for a person who fails to report an offence? a) Imprisonment up to 5 years with a fine b) Up to 6 months with a fine c) Up to 2 years with a fine d) Fine only 3) To whom is the POCSO legislation related? a) Women b) Under-age girls c) Old people d) Gender neutral 4) Which of the following is not included under the POCSO Act? a) Sexual assault (penetrative and non-penetrative) b) Sexual harassment c) Child pornography d) Child kidnapping 5) Who can report an offence under POCSO? a) Anyone b) Treating doctor c) Only parents d) Higher official 6) Sexual assault becomes aggravated when: a) Offence is committed by a person of trust or authority b) Child is below 12 years c) Committed by a public servant, police officer, or hospital staff d) All of the above 7) Punishment for child pornography under the POCSO Act: a) 5 years, as well as a fine (may extend to 2-3 years) b) 3 years, as well as a fine (may extend to 2-3 years) c) 2 years, as well as a fine (may extend to 2-3 years) d) 10 years as well as a fine 8) The statement of the child should be recorded in the presence of: a) Magistrate/police, parents, or someone the child trusts b) Only parents c) Collector d) Doctors 9) When should the child be medically examined? a) After 24 hours of the report b) As soon as possible c) After formal complaint recording d) After court permission 10) The cases under POCSO should be disposed of by the court within: a) 2 years b) 3 years c) 1 year d) 6 months
